# Automatic localization and segmentation of focal cortical dysplasia in FLAIR‐negative patients using a convolutional neural network

**DOI:** 10.1002/acm2.12985

**Published:** 2020-08-18

**Authors:** Cuixia Feng, Hulin Zhao, Yueer Li, Junhai Wen

**Affiliations:** ^1^ Department of Biomedical Engineering School of Life Science Beijing Institute of Technology Beijing China; ^2^ Sixth Medical Center of PLA General Hospital Beijing China

**Keywords:** convolutional neural network, focal cortical dysplasia, FLAIR, localization, segmentation

## Abstract

**Purpose:**

Focal cortical dysplasia (FCD) is a common cause of epilepsy; the only treatment is surgery. Therefore, detecting FCD using noninvasive imaging technology can help doctors determine whether surgical intervention is required. Since FCD lesions are small and not obvious, diagnosing FCD through visual evaluations of magnetic resonance imaging (MRI) scans is difficult. The purpose of this study is to detect and segment histologically confirmed FCD lesions in images of normal fluid‐attenuated inversion recovery (FLAIR)‐negative lesions using convolutional neural network (CNN) technology.

**Methods:**

The technique involves training a six‐layer CNN named Net‐Pos, which consists of two convolutional layers (CLs); two pooling layers (PLs); and two fully connected (FC) layers, including 60 943 learning parameters. We employed activation maximization (AM) to optimize a series of pattern image blocks (PIBs) that were most similar to a lesion image block by using the trained Net‐Pos. We developed an AM and convolutional localization (AMCL) algorithm that employs the mean PIBs combined with convolution to locate and segment FCD lesions in FLAIR‐negative patients. Five evaluation indices, namely, recall, specificity, accuracy, precision, and the Dice coefficient, were applied to evaluate the localization and segmentation performance of the algorithm.

**Results:**

The PIBs most similar to an FCD lesion image block were identified by the trained Net‐Pos as image blocks with brighter central areas and darker surrounding image blocks. The technique was evaluated using 18 FLAIR‐negative lesion images from 12 patients. The subject‐wise recall of the AMCL algorithm was 83.33% (15/18). The Dice coefficient for the segmentation performance was 52.68.

**Conclusion:**

We developed a novel algorithm referred to as the AMCL algorithm with mean PIBs to effectively and automatically detect and segment FLAIR‐negative FCD lesions. This work is the first study to apply a CNN‐based model to detect and segment FCD lesions in images of FLAIR‐negative lesions.

## 1 INTRODUCTION

Focal cortical dysplasia (FCD) is a malformation of cortical development and one of the most common causes of intractable epilepsy, as defined by Taylor and his colleagues in 1971.^1^ Currently, the main treatment for drug‐resistant epilepsy is surgery, and magnetic resonance imaging (MRI) is usually an ideal tool for detecting FCD. Mainly, MRI features of FCD include focal cortical thickening, fuzziness between gray matter (GM) and white matter (WM), cortical/subcortical WM hyperintensity on T2‐weighted imaging (T2WI)/fluid‐attenuated inversion recovery (FLAIR), widened gyri, and abnormal sulci.^2^ Currently, three main conventional methods for detecting epileptic foci exist: the voxel‐based morphometry (VBM) algorithm;^3^, ^4^ surface‐based morphometry (SBM) algorithm;^5^, ^6^, ^7^ and postprocessing method,^8^, ^9^, ^10^ which is based on pixel feature extraction. Although these techniques perform extremely well, the VBM technique employs a few neurological features and is sensitive to artifacts, and the SBM technology has high computational complexity because of the three‐dimensional (3D) surface reconstruction.

In 1998, LeNet‐5, which is based on the convolutional neural network (CNN) proposed by Lecun et al.,^11^ was successfully applied in handwritten character recognition. In 2012, AlexNet^12^ won an image classification competition by using the large database ImageNet. After AlexNet, a variety of new CNN models, such as the Visual Geometry Group (VGG) of the University of Oxford, Google's GoogLeNet,^13^ Microsoft's Residual Networks (ResNet),^14^ and the Dense Convolutional Network (DenseNet),^15^ were proposed. Preliminary progress in understanding CNNs has been achieved. Class activation mapping (CAM)^16^ and gradient‐weighted CAM (Grad‐CAM)^17^ can restore the location function of the convolutional layer (CL) and identify the locations of unsupervised feature regions. The deconvolution network (Deconvnet)^18^ and guided backpropagation (GBP) can visualize the feature extraction of each CL. The activation maximum (AM)^19^ can understand how neural networks recognize features by generating a pattern image.

CNNs are highly accurate in object detection and segmentation, such as pedestrian detection,^20^ action detection,^21^ object detection,^22^ and saliency detection.^23^ Current research focuses on natural images, and research in medicine includes tumor detection^24^ and tumor segmentation.^25^ All of these examples show that CNNs are excellent algorithms in image recognition and segmentation. In epilepsy, CNNs have been employed to detect abnormal electroencephalogram (EEG) signals in epilepsy.^26^, ^27^, ^28^ We identified two studies on FCD focus recognition and location using CNNs; these studies obtained effective results. The first study employed a CNN model to automatically segment FCD lesions in FLAIR images.^29^ The second study applied a deep CNN to classify FCD and non‐FCD patches in a T1 image.^30^

The current research is based mainly on either T1 images or T1 images combined with T2 or FLAIR images, and feature extraction is based on artificial extraction. The common findings of FCD are cortical or subcortical hyperintensities, which are especially observed in FLAIR images. Manually extracting features to distinguish FCD pixels from normal pixels is difficult. In this study, we propose a novel automated FCD detection and segmentation technique, which is referred to as activation maximization and convolutional localization (AMCL). This technique, which is based on an advanced CNN, is the first technique to automatically analyze the features of FCD lesions and locate them in FLAIR‐negative epilepsy patients. This technique is noninvasive, effective, highly accurate, and inexpensive and can not only alleviate the problem of uncertain factors, such as doctors' subjective experience and judgment of misdiagnosis, but also assist doctors in implementing effective preoperative evaluation and completely removing the lesion area during the operation to completely cure epilepsy.

## 2 MATERIALS AND METHODS

### 2.A Materials

Subjects: we selected 34 FLAIR images from 19 patients [average age ± standard deviation (SD) = 24 ± 10; seven males, 12 females] between 2012 and 2016. All of the data were provided by the Sixth Medical Center of PLA General Hospital (Haidian District, Beijing, China). All analyses were ethical, and this study was approved by the Sixth Medical Center of PLA General Hospital Institutional Ethics Committee.

The lesion tissues of all drug‐refractory epilepsy patients were resected, and the pathological results showed FCD. The surgery was based on strong clinical and EEG information. The ground truth FCD regions were semiautomatically retrospectively segmented depending on the resection by an experienced epileptologist with 16 yr of experience in performing epilepsy operations. The FLAIR images included two kinds of images: FLAIR‐positive lesion images (P1–P7) and FLAIR‐negative lesion images (P8–P19). Lesions were observed by the epileptologist in 16 FLAIR‐positive lesion images of seven patients before their operations, and lesions were not observed by the epileptologist in 18 FLAIR‐negative lesion images of 12 patients before their operations. When multiple images of a patient are selected, images in different directions are selected instead of images in continuous layers. The lesions were located in the frontal lobe (of three patients), occipital lobe (of four patients), parietal lobe (of four patients), temporal lobe (of 11 patients), and left inferior precuneus (of one patient). The FCD classification is based on the classification scheme for cortical development.^31^ Detailed pathological information is shown in Table 1.

**Table 1:** Patient details.

P	Onset age	Sex	Surgical resection region	FCD type	MR pulse sequence	MRI acquisition parameters (TR/TE/FA/DFOV/Field/ST)
P1	29	M	Right occipital lobe	II b	TSE	5000 ms/396 ms/120°/240 mm/3 T/1.5 mm
P2	7	F	Right occipital lobe	I b	FSE	9002 ms/126 ms/90°/–/1.5 T/2 mm
P3	23	M	Left parietal and occipital lobe	I b	TSE	5000 ms/396 ms/120°/195 mm/1 T/1 mm
P4	6	F	Right frontal lobe	II a	FSE	5000 ms/396 ms/120°/240 mm/3 T/1.5 mm
P5	20	F	Right parietal and occipital lobe	II a	FSE	9602 ms/141.7 ms/90°/240 mm/3 T/5 mm
P6	16	F	Right parietal cortex	II b	FSE	9002 ms/126 ms/90°/–/3 T/1.5 mm
P7	18	M	Left inferior precuneus	II b	FSE	9002 ms/126 ms/90°/–/3 T/1.5 mm
P8	24	M	Left temporal lobe	II b	FSE	9002 ms/133 ms/90°/240 mm/1.5 T/4 mm
P9	34	M	Left temporal lobe	II a	FSE	9602 ms/145.24 ms/90°/240 mm/3 T/5 mm
P10	27	F	Right temporal lobe	II a	FSE	9002 ms/133 ms/90°/240 mm/1.5 T/5 mm
P11	12	F	Left temporal lobe	III a	FSE	9002 ms/133 ms/90°/240 mm/1.5 T/4 mm
P12	27	F	Right temporal lobe	II a	FSE	9002 ms/127.5 ms/90°/220 mm/1.5 T/5 mm
P13	24	M	Right frontal lobe	I b	FSE	9602 ms/146.33 ms/90°/240 mm/3 T/5 mm
P14	24	F	Right temporal lobe	II b	FSE	9602 ms/141.86 ms/90°/240 mm/3 T/5 mm
P15	27	M	Left temporal and parietal lobe	II b	FSE	9602 ms/146.34 ms/90°/240 mm/3 T/5 mm
P16	27	F	Right temporal lobe	I b	TSE	9602 ms/146.94 ms/90°/240 mm/3 T/5 mm
P17	40	F	Right temporal lobe	III b	TSE	5000 ms/396 ms/120°/240 mm/3 T/1.5 mm
P18	32	F	Left temporal lobe	II a	TSE	5000 ms/396 ms/120°/240 mm/3 T/1.5 mm
P19	46	F	Left frontal and temporal lobe	II b	TSE	5000 ms/396 ms/120°/240 mm/3 T/1.5 mm

M (male), F (female), FSE (fast spin echo), TSE (turbo spin echo), TR (repetition time),TE (echo time), FA (flip angle), DFOV (displayed field of view), and ST (slice thickness).

### 2.B Methods

This study developed an AMCL method that consists of five steps: (a) preprocessing the image, (b) designing and training a CNN, (c) obtaining a series of pattern image blocks (PIBs) by using the trained CNN, (d) using the mean PIB with convolution to locate and segment the lesion area, and (e) quantitatively evaluating the performance of the method. PyTorch was utilized to implement the proposed method. All experiments were performed on a Dell computer [Intel(R) Xeon(R) CPU E5‐1607 v3 @3.10 GHz, 16 GB RAM and NVIDIA]. To perform a comparison with the Grad‐CAM localization method, a simple procedure is implemented at the end of the proposed method.

#### 2.B.1 Image preprocessing

**Fig. 1:**
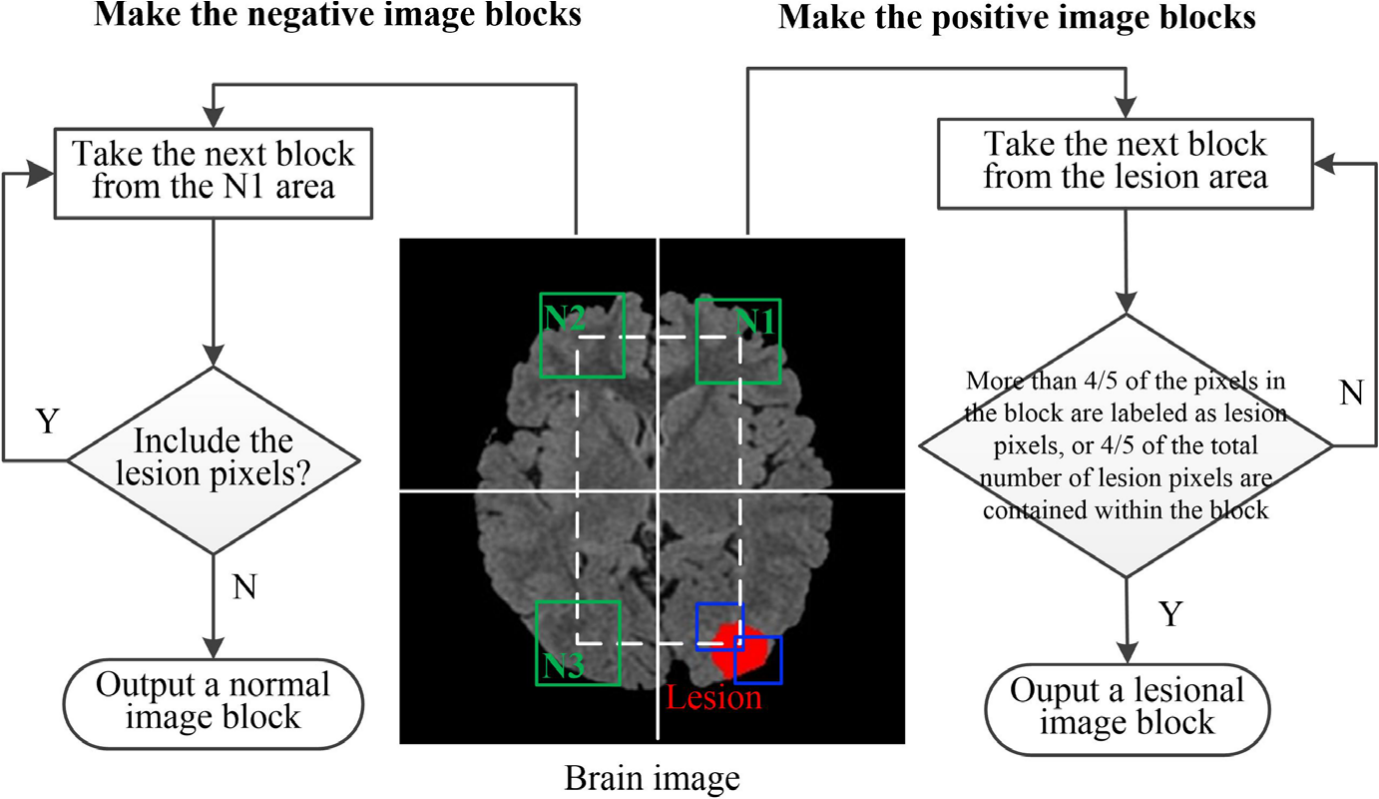
Constructing the dataset.

Training a CNN requires many training data; we employed a limited number of cases to construct sufficient training data. The dataset construction process is shown in Fig. 1. The FCD regions were manually marked by an epileptologist based on the operation location, as shown in the red area in Fig. 1. First, we extracted the brain region from the original image to perform the skull‐stripping operation, resized the brain image to a uniform resolution of 256 × 256 pixels by using bilinear interpolation, and normalized the brain image to a zero mean with a unit variance. Second, we selected 28 × 28 pixel square blocks from the brain image as the training data, in which the positive image blocks were obtained from the lesion area (as shown in the right part of Fig. 1) and the negative image blocks were obtained from the normal area (as shown in the left part of Fig. 1).

The details of the training set are described as follows: first, we selected a 28 × 28 block from the first pixel in the upper left corner of the lesion area, which was manually labeled in the red area as the center point of the block, as shown by the blue rectangular block in Fig. 1; second, we determined whether the block could be considered to be a lesion image block. If more than four‐fifths of the pixels in the block were labeled as lesion pixels or four‐fifths of the total number of lesion pixels were contained within the block, the block was considered to be a lesion image block and was output. If these conditions were not satisfied, the block window continued to be slid to the right or down to extract the next block for determination until each pixel in the lesion area was traversed as the center of the block. The number of all lesion image blocks obtained from the lesion area was calculated as the number of positive image blocks.

The normal image blocks were symmetrical with the center of the image, and the selected areas are N1, N2, and N3. To ensure that the number of positive image blocks is equal to the number of negative image blocks, we selected one‐third of the negative image blocks from the N1, N2, and N3 regions. First, we selected a 28 × 28 block from the N1 area. If the block has no lesion pixels, a normal image block is output. Otherwise, we continue to slide the block window to the right or down to remove the next block for evaluation. In selecting negative image blocks, we ensure that no lesion pixel exists in the symmetrical region, and if a lesion pixel exists, we exclude the block. Second, to increase the diversity of the image blocks, the original image was rotated every 24° to collect the image blocks, as in the previous procedure. Finally, we obtained 126 684 negative image blocks and 148 428 positive image blocks as the input of the network; the total number of image blocks included 69 224 negative image blocks and 77 868 positive image blocks from the FLAIR‐positive lesion images and 57 460 negative image blocks and 70 560 positive image blocks from the FLAIR‐negative lesion images. The number of positive and negative image blocks is approximately 1:1, which satisfies the requirements for training the network.

#### 2.B.2 Designing and training of the CNN

**Fig. 2:**
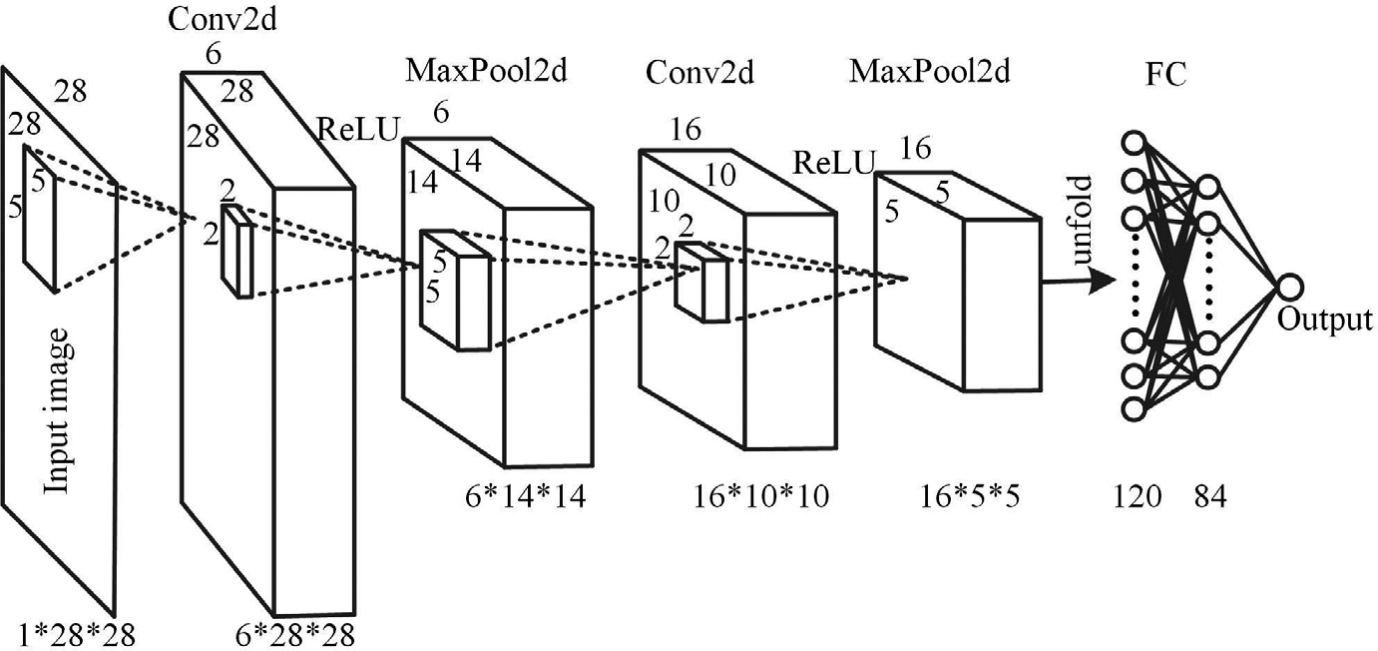
Structure diagram of the six‐layer convolutional neural network.

A complete CNN includes mainly the model structure definition, loss function selection, optimizer selection, and visual analysis of the final model. The CNN generally consists of an input layer, multiple CLs, multiple downsampling layers [or pooling layers (PLs)], a fully connected (FC) layer, and an output layer. The CL is the core layer of the CNN. Each filter traverses the input image and generates a two‐dimensional (2D) activation function image that is referred to as the feature map (FM). The hyperparameters include the number of convolutional kernels K, kernel size F, stride S, and padding zero P. The PL includes the averaging operation (AVG) and maximization operation (MAX). The hyperparameters include the kernel size F and stride S. In the last layer of the entire network, the FC layer is completely connected with the values of all the activation functions of the previous layer. A structure diagram of the CNN designed in this work is shown in Fig. 2.

Net‐Pos consists of two CLs, two PLs, and two FC layers, including 60 943 learning parameters. The input image of the network is D1×W1×H1=1×28×28. The selected parameters of the first layer (Conv2d) are F=5, S=1, P=2, and K=6; and the image size becomes 6 × 28 × 28. The parameters selected for the second layer (MaxPool2d) are F=2 and S=2, and the image size becomes 6 × 14 × 14. The parameters selected for the third layer (Conv2d) are F=5, S=1, P=0, and K=16; and the image size becomes 16 × 10 × 10. The fourth layer (MaxPool2d) is the same as the second layer, and the image becomes 16 × 5 × 5. The 16 × 5 × 5 image is unfolded into a column vector of 1 × 400 as the input of the FC layer. The fifth layer contains 120 neurons, and the sixth layer contains 84 neurons. A probability is output. Each CL is connected with an ReLU activation function, which helps the network acquire nonlinear features and avoid gradient dissipation. The output of the network represents the probability that an image shows disease. If the output is >0.5, the image is considered to show disease, and if the output is <0.5, the image is considered normal. The loss function is shown in Eq. (1):

(1) $$L\left(\theta \right)=‐(y\cdot \log\left(h_{\theta }\left(X\right)\right)+\left(1‐y\right)\cdot \log\left(1‐h_{\theta }\left(X\right)\right))$$

where $h_{\theta }\left(X\right)$ is the actual output of the network and y∈{0,1} is the real label.

We establish the sample set $\left\{\left(\left(X^{1},y^{1}\right),\cdots \left(X^{m},y^{m}\right)\right\}\right)$, which contains m input images X. The overall cost function is defined as shown in Eq. (2):

(2) $$J\left(\theta \right)=\left[\frac{1}{m}\sum_{i=1}^{m}L(\theta )\right]=\left[\frac{1}{m}\sum_{i=1}^{m}\left(‐(y\cdot \log\left(h_{\theta }\left(X\right)\right)+\left(1‐y\right)\cdot \log\left(1‐h_{\theta }\left(X\right)\right))\right)\right]$$

To minimize the overall loss function, J(θ), the stochastic gradient descent with momentum (SGDM) optimizer, is employed in the proposed technique. The weights update the network parameters, as shown in Eq. (3):

(3) $$\theta_{n+1}=\theta_{n}‐lr\cdot \rho \cdot \left(\theta_{n}‐\theta_{n‐1}\right)‐lr\frac{\partial }{\partial \theta_{n}}J\left(W,b\right)$$

where θ is the parameter vector, n is the iteration number, ρ is the momentum, and lr is the initial learning rate. In our work, ρ=0.9, and lr=0.01.

After several iterations, the network tends to converge, and the loss function tends to zero, thereby indicating that the network training is completed; then, image classification or localization can be carried out.

In the subsequent image recognition, PIBs are used for template convolution, so we employed two sets of data to train two networks to obtain the optimal PIBs. The structures of the two networks are the same, but the data utilized in the training are different. The first network, which is named Net‐Pos‐Neg, was trained by using all of the data (126 684 negative image blocks and 148 428 positive image blocks) obtained from the FLAIR‐positive and FLAIR‐negative lesion images. The second network, which is named Net‐Pos, was trained by using the data (69 224 negative image blocks and 77 868 positive image blocks) obtained from the FLAIR‐positive lesion images. The whole dataset was divided into training and testing sets based on the 80–20% training rule, and the training and testing sets were obtained from unique patients.

#### 2.B.3 Obtaining the PIBs

The fundamental concept of AM adopts the gradient descent to iteratively update the pixels of the input image to maximize the average activation of a specific neuron of the specific FM and obtain the PIB. AM includes mainly three steps: (a) inputting a random or noise image into the trained CNN in evaluation mode; (b) calculating the gradients of a specific neuron in a specific layer with respect to the noise image to maximize the neuron; and (c) iteratively updating each pixel in the noise image to maximize the activation of the neuron and obtain a final image, which is referred to as the pattern image.

**Fig. 3:**
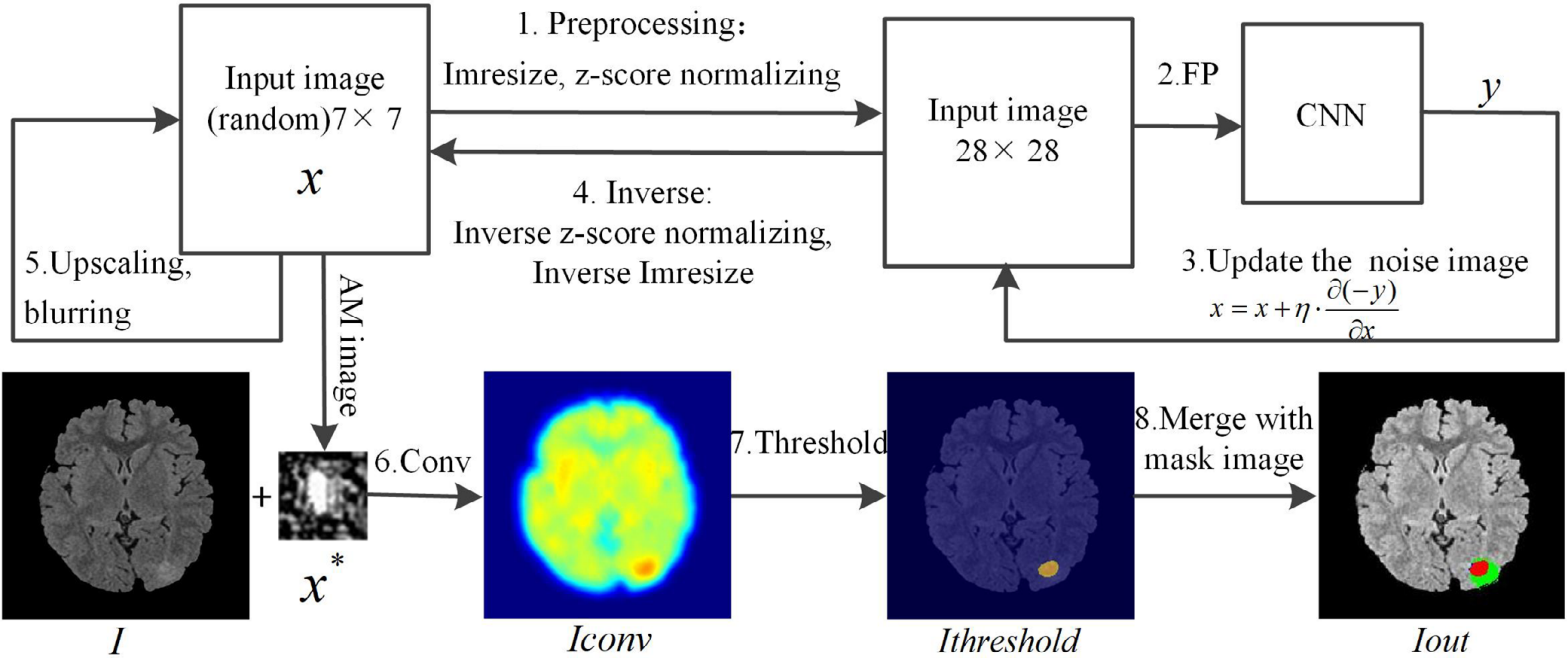
Flowchart of the activation maximization and convolutional localization.

The process of obtaining the PIB is shown in steps one to five in Fig. 3. The optimization process of the PIB applies Eq. (4):

(4) $$x^{*}=\underset{x}{\arg\max a_{i,l}\left(\theta ,x\right)}$$

where $x^{*}$ is the final image (or PIB), $a_{i,l}$ denotes the activation value of the i neuron in layer l, x is the random image of the input, and θ denotes the parameters of the network.

Step 1: Image block preprocessing. First, the input image was set to a random image block of size 7 × 7 and then resized to the CNN input size of 28 × 28. Second, z‐score normalization was performed.

Step 2: Forward propagation. The preprocessed image block was input into the trained CNN for forward propagation, and the output is the probability that the image block contains disease.

Step 3: Update the noise image block. We calculated the PIBs of the output neurons. The probability of the output neuron of the CNN was employed as a loss function of backpropagation. The gradient of the loss function to the noise image block was calculated. The noise image block was updated according to the gradient descent algorithm. The updated equation is shown in Eq. (5):

(5) $$x=x+\eta \cdot \frac{\partial (a_{i,l}\left(\theta ,x\right))}{\partial x}$$

where $a_{i,l}\left(\theta ,x\right)=y$ or $a_{i,l}\left(\theta ,x\right)=‐y$; then, steps 2 and 3 are repeated 20 times.

Step 4: Inverse operation. The iterated image block was inversely operated by normalizing and resizing the z‐score, and the 7 × 7 image block was obtained.

Step 5: Upscaling and blurring. The 7 × 7 image block was upscaled with a scale factor of 1.2, and then, the image block was smoothed (blurring size = 2); steps 1–4 were repeated eight times to obtain the final noise‐free image, which is also referred to as PIB $x^{*}$.

In this study, we calculate the PIB of the output neuron. We set the output neuron $a_{i,l}\left(\theta ,x\right)=‐y$. The output probability is one when $x^{*}$ is input into the trained CNN, thus indicating that $x^{*}$ 100% is the lesion image block. Conversely, we set $a_{i,l}\left(\theta ,x\right)=y$, and the output probability of $x^{*}$ input into the CNN is zero, indicating that $x^{*}$ 100% is a normal image block. When we input the random image block into the two networks (Net‐Pos‐Neg and Net‐Pos) and set $a_{i,l}\left(\theta ,x\right)=‐y$ and $a_{i,l}\left(\theta ,x\right)=y$, two kinds of PIBs are obtained from the two networks, as shown in Fig. 5.

If the 28 × 28 random image was input directly for iteration, the high‐frequency information would be dominant in the $x^{*}$ PIB, and the boundaries of the PIBs would change substantially. To reduce the high‐frequency information and smooth the image boundaries in the PIBs, the pixels are optimized from the low resolution of 7 × 7, and then, the image block is upscaled. Low‐frequency, high‐resolution, noise‐free PIBs can be obtained by a sufficient number of iterations. To further reduce the high‐frequency information, image smoothing was performed in Step 5.

#### 2.B.4 Convolutional localization and segmentation

Step 6: Template the convolutional operation. We employed the mean PIB from four runs of AM to perform a traversal pattern‐matching convolutional operation to locate and segment the lesion to be recognized in the image. The mean $x^{*}$ was slid along image I with stride one for recognition, and the convolution sum of the whole image was traversed. The calculation equation is shown in Eq. (6):

(6) $$Iconv(i,j)=sum(conv\left(I\left(i‐13:i+14,j‐13:j+14\right),x^{*}\right))$$

where I(i‐13:i+14,j‐13:j+14) extracts the 28 × 28 block from the original image to be recognized and i and j are the length and width of the image, respectively.

Step 7: Threshold. Select the lesion area from the convolved image with an appropriate threshold and set any value of <0.9·max(Iconv) in the Iconv image to zero. This threshold is the most appropriate value obtained via the receiver operating characteristic (ROC) curve.

Step 8: Display. The localization result, original image, and ground truth image, which are hand labeled by the epileptologist, were merged into one color image to show the lesion. The color area is labeled manually by an epileptologist in red and green, and the detected area is manually labeled in red and blue.

The whole process is shown in Fig. 3.

Due to the limited number of patients, we employed the leave‐one‐out cross‐validation (LOOCV) strategy, in which each patient of a set of N patients is classified by using a classifier that is trained on the remaining N − 1 patients to evaluate the proposed method. Because the Net‐Pos training is based on the image blocks extracted from the FLAIR‐positive lesion images, the LOOCV strategy is utilized in the location and segmentation of FLAIR‐positive lesion images. FLAIR‐negative lesion images belong to the new dataset for the Net‐Pos network; therefore, the mean PIB is directly applied to locate and segment FLAIR‐negative lesion images.

### 2.C Grad‐CAM localization

**Fig. 4:**
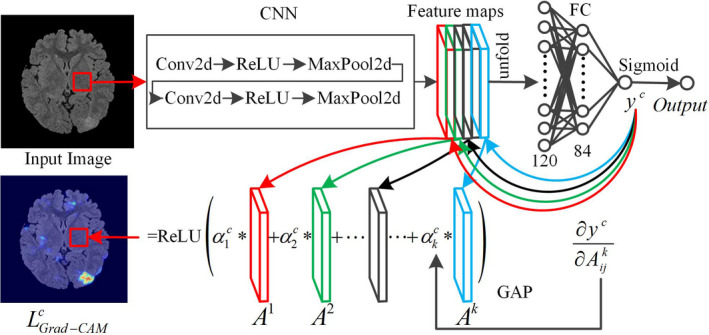
Flowchart of the Grad‐class activation mapping.

Grad‐CAM^17^ is a visualization method that is typically employed to identify the key feature area that most greatly affects the classification results. This method employs the gradient information and the last CL of the CNN to understand the importance of each neuron for a decision of interest. Previous studies have indicated that the CL in the CNN has the function of locating the target. Once the FMs of the last layer are unfolded into one column vector of FC layers for classification, the function of locating the target of the CNN loses. In this study, we applied the idea of identifying FCD lesions in the images. We combined the last FMs with the gradient information of the output and the last layer of the FMs to identify the key feature of the FCD area. The flowchart of Grad‐CAM is shown in Fig. 4.

Step 1: We consider a 28 × 28 block from the original image as the input of the CNN and propagate the block forward to the last FM of the CL. The global average pooling (GAP) technique^32^was employed to calculate the FM weight of category c, as shown in Eq. (7). The gradient of $y^{c}$ to FM $A^{k}$ was calculated, and then, the gradients were averaged to obtain the weight $\alpha_{k}^{c}$:

(7) $$\alpha_{k}^{c}=\frac{1}{Z}\sum_{i}\sum_{j}\frac{\partial y^{c}}{\partial A_{\mathrm{ij}}^{k}}$$

where $y^{c}$ is the value of category c before the last activation function; $A^{k}$ is the k‐th FM of the last layer of the CL; i and j are the pixel sizes of the length and the width, respectively, of the FM; and Z=i·j.

Step 2: The final mapping image $L_{Grad‐CAM}^{c}$ is obtained by the linear combination of $A^{k}$ and $\alpha_{k}^{c}$ and then by the ReLU activation function, as shown in Eq. (8). When Output>0.5, we perform backpropagation and obtain the $L_{Grad‐CAM}^{c}$. image:

(8) $$L_{Grad‐CAM}^{c}=\text{Re}LU\left(\sum_{k}\alpha_{k}^{c}A^{k}\right)$$

Combining the FMs with the gradient of the output to the FM can determine the difference between the lesion area and the normal area in the original image as much as possible and highlight the lesion area. The color region in the $L_{Grad‐CAM}^{c}$ image is the probability of recognizing the region as a lesion.

#### 2.D Quantitative evaluation

In this study, all images that need to be recognized are images with lesion areas. Because each patient's image is selected in different directions, the detection of each image can be considered subject‐wisely. We utilized the recall to subject‐wisely calculate the accuracy. To measure the robustness of the proposed method, the FCD detection performance was measured pixelwisely. The specificity, accuracy, recall, and precision were defined as follows: Specificity=TN/(TN+FP)·100, Accuracy=(TP+TN)/(TP+FP+TN+FN)·100, Recall=TP/(TP+FN)·100and Precision=TP/(TP+FP)·100. To evaluate the segmentation of FCD lesions, we adopted the Dice coefficient Dice=2·TP·100/(2·TP+FP+FN). TP and TN are the number of correctly detected FCD pixels and the number of normal pixels, respectively. FP and FN are the number of falsely detected FCD pixels and the number of normal pixels, respectively.

## 3 RESULTS

### 3.A The PIBs

**Fig. 5:**
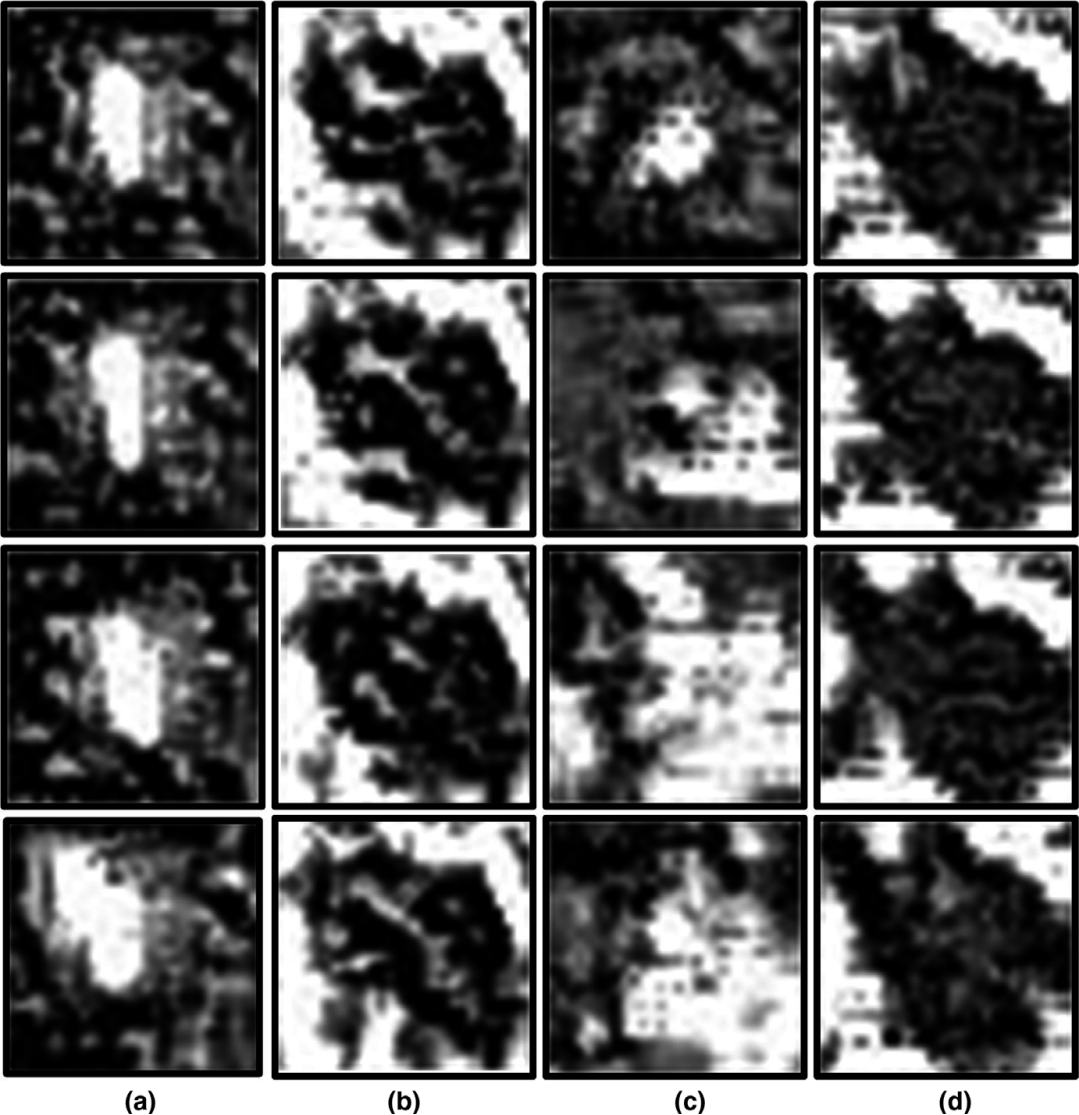
Pattern image blocks. Each row corresponds to a different iteration. (a) and (b) are obtained from Net‐Pos and represent image blocks similar to lesion image blocks and normal image blocks, respectively. (c) and (d) are derived from Net‐Pos‐Neg and represent image blocks similar to lesion image blocks and normal image blocks, respectively.

Four instances of the two kinds of PIBs obtained via Net‐Pos and Net‐Pos‐Neg are shown in Fig. 5.

### 3.B Localization results

**Fig. 6:**
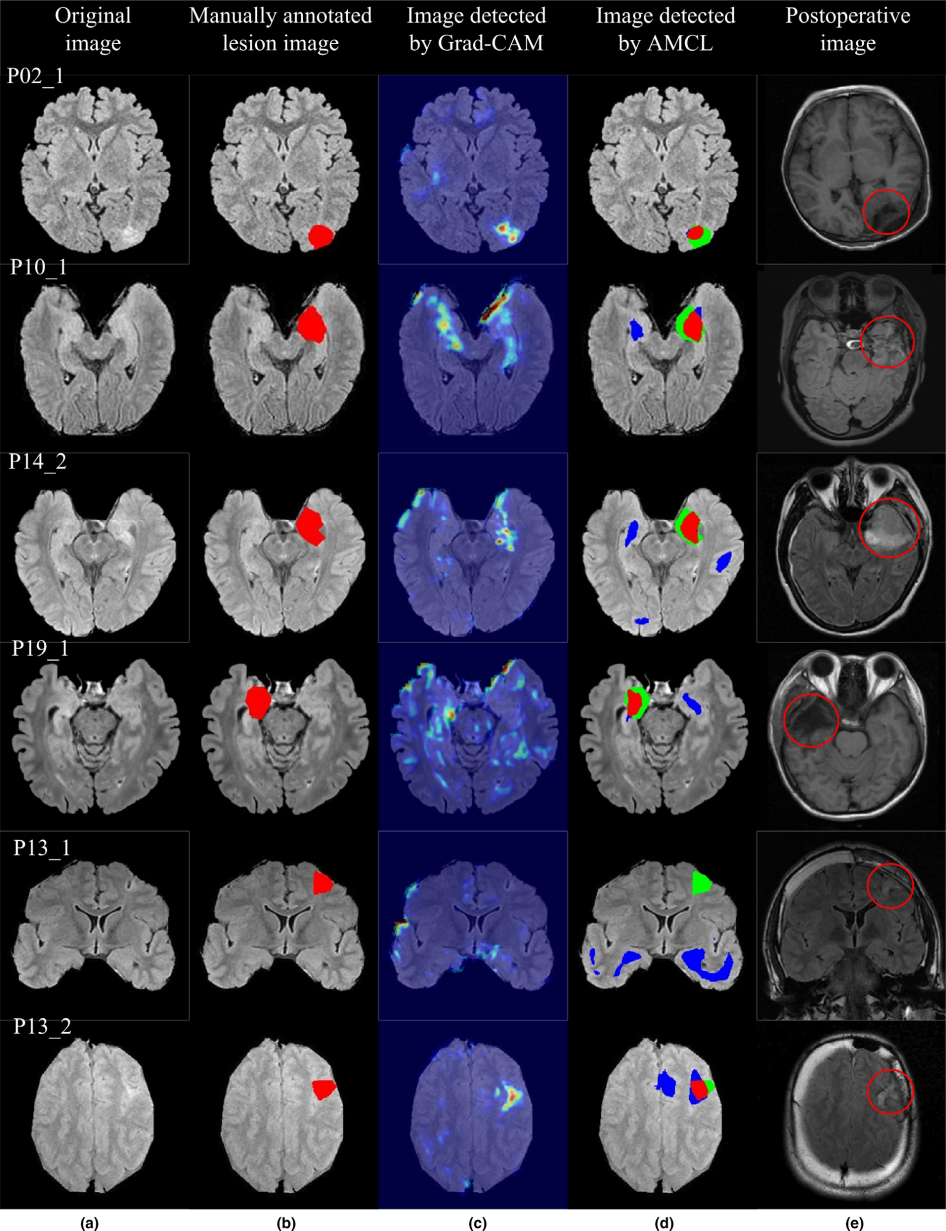
The detection results for patient P02, with a fluid‐attenuated inversion recovery (FLAIR)‐positive lesion, are shown in the first row. The detection results for three patients (P10, P14, and P19) with FLAIR‐negative lesions are shown in the second to fourth rows. For patient P13, the lesion is detected in the axial images but is undetected in the coronal images, as shown in the last two rows. (a) Original image. (b) Lesion area labeled as ground truth. (c) Localization results of Grad‐class activation mapping. (d) Localization results of activation maximization and convolutional localization. (e) Postoperative images.

The detection results of some images are shown in Fig. 6. A patient with a FLAIR‐positive lesion is shown in the first row, and four patients with FLAIR‐negative lesions are shown in the second to fifth rows.

The localization results of AMCL are shown in the fourth column. The red area denotes TP, the green area denotes FP, and the blue area denotes FN. The postoperative image after operation is shown in the fifth column, and the area in the circle is the area after operation.

### 3.C Quantitative results

A quantitative evaluation of the Grad‐CAM and AMCL is shown in Table 2. The recall was calculated subject‐wisely. The specificity, accuracy, recall, and precision were calculated pixel‐wisely. When the recall was >20, FCD lesions were considered. Although our purpose is to detect and segment the focus of the FLAIR‐negative lesion images, we still present the segmentation results of the FLAIR‐positive lesion images in the table, which is valuable.

**Table 2:** Quantitative evaluation of Grad‐class activation mapping (CAM) and activation maximum and convolutional localization (AMCL).

Algorithm	P/N	Subject‐wise	Pixel‐wise				
		Recall	Specificity	Accuracy	Recall	Precision	Dice coefficient
Grad‐CAM	P	93.75	98.90	98.54	58.66	38.17	58.58
	*N*	55.55	98.66	98.09	37.15	22.96	32.3
AMCL	P	100	99.57	99.15	59.44	55.44	71.18
	*N*	83.33	99.01	98.43	50.64	39.95	52.68

The table presents the evaluation of the location results by the two algorithms. Each cell shows the mean of all of the patient detection results. P represents the FLAIR‐positive lesion images, and N represents the FLAIR‐negative lesion images.

A comparison of the proposed and other current techniques is shown in Table 3. The location and segmentation results of Grad‐CAM are the results obtained by using the FLAIR‐negative lesion images in this study.

**Table 3:** Performance comparison with current techniques.

Related work	Method	Subject‐wise	Pixel‐wise		
		Recall	Recall	Precision	Dice coefficient
Pail et al.^3^	VBM	70	–	–	–
Ahmed et al.^6^	SBM	58	2.47	–	3.68
Bijay Dev et al.^29^	CNN	82.5	40.1	80.69	52.47
Wang, Huiquan et al.^30^	CNN	90	–	–	78
Selvaraju et al.^17^	Grad‐CAM	55.55	37.15	22.96	32.3
*Proposed*	*AMCL*	83.33	50.64	39.95	52.68

## 4 DISCUSSION

### 4.A The PIBs

A comparison of Figs. 5(a), 5(b), 5(c), and 5(d) shows that the Net‐Pos network has converged to the optimal network parameters, while the Net‐Pos‐Neg network has not. Figures 5(a) and 5(b) show two kinds of PIBs obtained by four iterations of Net‐Pos. The two kinds of PIBs are very similar with different random images input each time, thereby showing that the Net‐Pos network is stable and can obtain consistent PIBs through the network. Figures 5(c) and 5(d) show two kinds of PIBs obtained via four iterations of Net‐Pos‐Neg. We discovered that the PIBs changed greatly with different random images input each time. This finding shows that the Net‐Pos‐Neg network is unstable, and the image blocks that are similar to the lesion image blocks were undetected.

The comparison of Figs. 5(a) and 5(b) shows that that we have found that one kind of image is similar to lesion image blocks, and the other is similar to normal image blocks. The output probability is one when the PIBs of Fig. 5(a) are input into the CNN, thus indicating that these PIBs are the most similar to the lesion image blocks. The output probability is zero when the PIBs of Fig. 5(b) are input into the CNN, thus indicating that these PIBs are the most similar to normal image blocks. The PIBs most similar to the lesion image blocks in Fig. 5(a) are the blocks with a brighter central area and darker surrounding images. Therefore, if a block area extracted from the original image has a high similarity to these PIBs, we can consider this block to be a lesion image block.

For patients with FLAIR‐negative lesions, traditional visual analysis cannot detect the lesion areas, and it is not known which features are effective for identifying such a lesion. CNNs are capable of automatically learning the appropriate features from labeled training data without any human intervention, thereby avoiding the limitations of manual feature extraction. Currently, the main method of performing image recognition using CNN technology is to adopt the typical network structure; there is no mathematical proof or specification to determine the best network model, and the training of the network depends mainly on experience. Therefore, the novelty of the CNN used in this study does not lie in the construction of a new CNN model; instead, it lies in the ability to use a trained CNN to find suitable PIBs to identify FLAIR‐negative lesions in images by means of PIB template matching. This method can avoid the ineffectiveness of manual feature extraction and enable better identification of lesion areas.

### 4.B Localization results

The detection results for a patient with a FLAIR‐positive lesion are shown in the first row of Fig. 6. In the fourth column of Fig. 6, the larger the red area in the image is, the more accurate the results are. The results of AMCL show that the main areas are red and green with fewer blue areas, thus indicating that the detection results of the proposed method are accurate. The detection results of AMCL and Grad‐CAM are consistent, and the FLAIR‐positive lesion can also be detected by Grad‐CAM.

The detection results in the second to fourth rows of Fig. 6 show that Grad‐CAM is not effective, meaning that it is unable to detect the lesions, whereas AMCL can identify the FCD lesions. Current MR technology provides excellent detection for FCD lesions that occur in extratemporal locations but is unreliable for detecting most FCD lesions, which predominantly occur in the temporal lobe.^33^ The lesions of three patients occurred in the temporal lobe region, and the proposed method can still detect the focus area, thereby indicating the effectiveness of the algorithm. Detecting temporal lobe epilepsy is difficult mainly because the left and the right temporal lobe were usually very bright on FLAIR images, thus increasing the difficulty of localization. Although the proposed method can detect the location of the FCD lesion, some false‐positive areas existed. Therefore, further studies should consider how to remove these false‐positive areas.

The limiting results of one patient, P13, is a special case. The lesion was detected in the axial images but was undetected in the coronal images. The two contrasting images are shown in the last two rows of Fig. 6. The detection of the P13_1 coronal image failed completely, and the lesion area could be detected in the P13_2 axial image. These two images were obtained using the same series of MR parameters. The MR findings of the patient P13 showed atrophy of some gyri and abnormal sulci in the right frontal lobe. The main reasons for image detection failure for the P13_1 coronal image is the small intensity change between the FCD pixels and the adjacent normal pixel and the large interclass similarity between the FCD pixels and the surrounding normal region. In the P13_1 coronal image, only tiny highlighted areas exist around the sulcus area, which is overlooked. Therefore, some defects in the detection of FCD lesion areas are observed in a single‐direction slice of two‐dimensional images.

Currently, the two studies that use CNN technology to detect FCD lesions also process 2D images.^29^, ^30^ Theoretically, we could obtain the three‐dimensional (3D) pattern image block that is spatially correlated with the FCD disease by training a 3D CNN. After expanding to three dimensions, the number of parameters that need to be trained in the network will increase significantly; thus, numerous 3D training data will be required to make the network converge. Therefore, with an increase in the number of samples in the future, we will also consider 3D processing while considering neighborhood information in spatial processing, which should be more accurate in theory.

### 4.C Quantitative results

Table 2 shows the performance analysis of Grad‐CAM and AMCL. The results of Grad‐CAM for the two categories of input images are slightly inferior to the results of the AMCL algorithm. From a subject‐wise perspective, the recall results of AMCL for FLAIR‐positive lesion images and FLAIR‐negative lesion images are 100% (16/16) and 83.33% (15/18), respectively. We achieved a Dice coefficient of 52.68 with the manual labels for FLAIR‐negative lesion images; this finding suggests strong agreement with the output images.

Table 3 shows the performance comparison with other existing techniques. The method by Pail et al. 2012 belongs to the VBM method, which mainly enhances the FCD lesion area; the results of this method are subject‐wise and, therefore, lack voxel‐wise results.^3^ Ahmed et al. utilized the SBM method to analyze T1‐negative images, and the final segmentation effect is not ideal. Advanced CNN technologies work better than traditional techniques. Dev et al employed the popular U‐Net architecture and trained a fully CNN (FCN) for FCD lesion identification and segmentation by using only FLAIR images.^29^ Because the purpose of this study is to perform segmentation, better segmentation results were achieved. Wang, Huiquan et al. employed a deep CNN to classify the cortical FCD patches into FCD and non‐FCD patches with T1 images.^30^ The two studies did not mention whether the images were positive or negative. Compared with the results of the two CNN algorithms, the proposed AMCL in this study is slightly superior mainly because all the images in this study are negative lesion images, for which location and segmentation are difficult. In addition to this reason, the lesions segmented by the doctor may not be particularly accurate; any such inaccuracies may have reduced the results of the quantitative analysis. Therefore, the manual labeling of lesions needs to be further optimized. If we compare the detection results of the FLAIR‐positive lesion images, the proposed method is much better than the methods proposed by previous studies. The ratio of the number of FLAIR‐positive lesion images to the number of FLAIR‐negative lesion images is 16:18, thus indicating that the sample balance and results are desirable. All of the experimental results show that the proposed technique, AMCL, is very effective and can provide higher detection and segmentation accuracy for FLAIR‐negative patients.

## 5 CONCLUSION

In this study, first, we trained a six‐layer CNN named Net‐Pos. Second, AM was performed to identify the PIBs that are most similar to the lesion image based on the trained Net‐Pos. We explained what kind of image block was most easily considered to be the lesion image block based on the PIBs. A PIB is a kind of image block with a brighter central area and darker surrounding images. The proposed method presented in this study has been applied to 34 FLAIR images (including 18 FLAIR‐negative lesion images) of 19 patients. The experimental results show that the AMCL algorithm is very effective in locating and segmenting FCD lesions, thus providing doctors with suspicious areas of epileptogenic lesions.

## CONFLICT OF INTEREST

No conflict of interest.

## AUTHORS' CONTRIBUTIONS

Cuixia Feng mainly designed and implemented the algorithms and was a major contributor in writing the manuscript. Hulin Zhao mainly sorted out the patient data and manually marked the lesions. Yueer Li was mainly responsible for proofreading the article. Junhai Wen provided overall guidance and full participation. All authors read and approved the final manuscript.
